# Listening to mom in the neonatal intensive care unit: a randomized trial of increased maternal speech exposure on white matter connectivity in infants born preterm

**DOI:** 10.3389/fnhum.2025.1673471

**Published:** 2025-10-14

**Authors:** Katherine E. Travis, Melissa Scala, Virginia A. Marchman, Hua Wu, Cory K. Dodson, Lisa Bruckert, Molly F. Lazarus, Rocío Velasco Poblaciones, Kristen W. Yeom, Heidi M. Feldman

**Affiliations:** ^1^Division of Developmental-Behavioral Pediatrics, Department of Pediatrics, Stanford University School of Medicine, Stanford, CA, United States; ^2^Burke-Cornell Medical Research Institute, Department of Pediatrics, Weill Medical College, Cornell University, New York, NY, United States; ^3^Division of Neonatal and Developmental Medicine, Department of Pediatrics, Stanford University School of Medicine, Stanford, CA, United States; ^4^Department of Psychology, Stanford University, Stanford, CA, United States; ^5^Cognitive and Neurobiological Imaging Center, Stanford University, Stanford, CA, United States; ^6^Department of Radiology, Stanford University School of Medicine, Stanford, CA, United States

**Keywords:** preterm (birth), language development, white matter (WM), intervention, NICU (neonatal intensive care unit)

## Abstract

**Objective:**

Early speech experiences are presumed to contribute to the development of brain structures involved in processing speech. Previous research has been limited to correlational studies. Here, we conducted a randomized trial with neonates born preterm to determine whether increased exposure to maternal speech during NICU hospitalization is causally linked to structural white matter maturation.

**Study design:**

We enrolled 46 neonates born preterm (24–31 weeks gestational age). Participants were randomly assigned to receive increased (T: *n* = 21) or routine (C: *n* = 25) exposure to mother’s speech. The T-group heard 10-min audio recordings of their mothers reading a children’s story two times/hour between 10pm and 6am, increasing speech exposure by 2.67 h/day. The C-group did not hear recorded speech. At near-term-equivalent age, we obtained two high-angular resolution diffusion MRI (scan 1: *b* = 700, scan 2: *b* = 1500) and T1 relaxometry scans. We assessed mean diffusivity (MD), pre-registered primary outcome (NCT02847689), of the left and right arcuate fasciculus, tracts implicated in language processing. Secondary outcomes included fractional anisotropy (FA) and R1 (1/T1). We hypothesized that neonates randomized to the T-group would show evidence for increased maturation within the arcuate, indexed as decreased MD and increased FA and R1, compared to neonates in the C-group.

**Results:**

Groups were equivalent on medical and demographic variables. Linear mixed models demonstrated that compared to the C-group, the T-group demonstrated significantly lower MD in the left (scan 1: β = −0.11, Marginal R^2^ = 0.27; scan 2: β = −0.12, Marginal R^2^ = 0.33) but not right arcuate (scan 1: β = −0.06, Marginal R^2^ = 0.09; scan 2: β = −0.03, Marginal R^2^ = 0.01). The T-group also demonstrated significantly higher FA (scan 1 β = 0.02, Marginal R^2^ = 0.20; scan 2: β = 0.03, Marginal R^2^ = 0.31) and R1 (β = 0.02, Marginal R^2^ = 0.39) in the left but not right arcuate.

**Conclusion:**

Preterm neonates with increased maternal speech exposure showed more mature left arcuate microstructure, supporting a causal role of exposure to speech in brain development. Enhancing speech exposure in the NICU may benefit preterm children’s language outcomes.

## Introduction

Early speech experiences are critical for language learning and are thought to contribute to the development of brain structures involved in processing spoken language (Kuhl, 2004). However, evidence of causal linkages is currently lacking. Children born very preterm (<32 weeks gestational age) are at risk for experiencing delays in language development and subsequent language-based learning disabilities (Nguyen et al., 2018). Adverse language outcomes in children born preterm have been linked to alterations in structural brain development (Back and Miller, 2014; Volpe, 2009), particularly changes in the microstructure of white matter circuits (Stipdonk et al., 2018). Brain development in preterm children may be affected by experiential factors, such as reduced exposure to maternal speech from spending multiple weeks to months in the neonatal intensive care unit (NICU) (Inder et al., 2023; Ment and Vohr, 2008). *In utero*, fetuses experience 2–3 times more speech from their mothers than do preterm infants at equivalent developmental ages (Monson et al., 2023). Interventions designed to enrich speech experiences in the NICU have shown positive benefits to short-term health outcomes among neonates born preterm (Filippa et al., 2017). Studies have yet to establish whether modifying speech experiences in the NICU can impact brain development in structures directly linked to language abilities in adults and children, including those born preterm.

Here, we performed a randomized controlled study to assess causal links between an intervention to increase exposure to spoken language and development of white matter circuits in infants born very preterm. To supplement speech experiences in the NICU, babies in the treatment group were played audio recordings of their mothers reading a children’s storybook and babies in the control group did not hear the audio recordings. We used recorded speech to control the timing and amount of speech exposure. Recorded speech has the additional empirical benefit of assuring a consistent source of input, helping to fill gaps in exposure that are difficult to address with live parental speech. Live speech can require interventions to educate parents to increase reading at bedside and spontaneous talk (Mayne et al., 2022; McGowan et al., 2024) and long periods of parental presence at bedside, something many families cannot sustain during prolonged hospital stays. We assessed intervention effects on measures of white matter microstructure acquired with diffusion MRI (dMRI) scans at near-term-equivalent age, prior to hospital discharge. We predicted that white matter would demonstrate sensitivity to the intervention based on evidence for myelin plasticity in animal models (Purger et al., 2016; Xin and Chan, 2020) and white matter responsivity to behavioral and language interventions in children and adults (Huber et al., 2018; Keller and Just, 2009; Scholz et al., 2009). We chose mean diffusivity (MD), a metric from dMRI, as the primary outcome measure for the following reasons. (1) MD has been shown to reflect age-related changes during development (Lebel et al., 2012; Yeatman et al., 2014) (2) MD is sensitive to behavioral interventions on a rapid timescale (Sagi et al., 2012) (3) MD is a continuous measure and thus may afford greater sensitivity to white matter plasticity compared to scalar diffusion tensor imaging metrics, such as fractional anisotropy (FA).

We focused on white matter tracts of the left and right arcuate fasciculus. These white matter pathways, and particularly the left arcuate, are known to be involved in speech and language processes. Multiple converging lines of evidence implicate fibers within the arcuate fasciculus in language processing. The arcuate connects the inferior parietal and posterior temporal cortices, regions implicated in speech-sound recognition, with inferior frontal cortices, regions implicated in motor-speech processes. Communication between these regions has been considered critical for conveying information relevant for both the perception and production of spoken language (Catani et al., 2005; Fedorenko et al., 2024; Glasser and Rilling, 2008; Ivanova et al., 2021; Poeppel and Hickok, 2004; Poeppel et al., 2016; Saur et al., 2008). We hypothesized that neonates randomized to the treatment group would show decreased MD within the arcuate, evidence of increased maturation, compared to neonates in the control group. Such findings would advance our understanding of how early experiences contribute to structural brain development. In addition, if successful, increasing exposure to speech via audio recordings among infants born preterm could serve as an inexpensive and scalable intervention to support recovery from alterations in brain development related to preterm birth and the NICU experience.

## Materials and methods

### Study design

This study was a prospective, parallel-group, randomized control trial, performed in a single neonatal intensive care nursery at the Lucile Packard’s Children’s Hospital (LPCH) Stanford University in Palo Alto, California.

### Participants

Female and male infants were eligible to enroll if they were born between 24-0/7 and 31-6/7 weeks gestational age (GA). Infants were cared for at a level IV NICU with an open bay design. We recorded sex, gestational age at birth, and birthweight as indicated in the electronic medical record. Infants were excluded from participation if they had conditions likely to impact brain development and therefore confound study findings, including: (1) congenital anomalies or recognizable malformation syndromes, (2) serious neurological conditions, including active seizure disorders, history of central nervous system infections or hydrocephalus, intraventricular hemorrhage grades III-IV, or cystic periventricular leukomalacia, (3) surgical treatment of necrotizing enterocolitis, (4) small for gestational age (<3rd percentile) and/or intra-uterine growth restriction, (5) twin-to-twin transfusion. Infants were also excluded if they were likely to be transferred from LPCH NICU to alternate care facility prior to 36 weeks postmenstrual age or the pre-discharge MRI scan, or if they had major sensori-neural hearing loss that would limit their ability to respond to the auditory based intervention. Families of eligible infants were approached once an infant was determined by clinical staff to be medically stable which typically coincided with infants’ transition to a step-down unit, the Packard Intermediate Care Nursery (PICN), and sufficient cardiorespiratory stability to tolerate developmental care activities including recorded auditory-speech exposure per unit protocols (Byrne et al., 2024). Parents of enrolled participants gave written informed consent before randomization.

### Randomization

Enrollment was performed by a study research coordinator, with assistance from the principal investigator (KET). Participants were randomly allocated to either the Treatment (T, speech exposure) or the Control (C, standard of care) group by the principal investigator using a minimization algorithm by Pocock and Simon (1975) implemented in the R statistical software package. Randomization was stratified for gestational age at birth (24-0/7–27-6/7 weeks or 28-0/7–31-6/7 weeks), to control for potential developmental differences in response to the intervention, and for socioeconomic status (SES) (low versus high SES; indexed as income status from public versus private insurance) to control for potential differences in language outcomes affected by SES factors. To obtain a continuous measure of SES, we collected a parent-report measure of SES, the Hollingshead four-factor index, which combines both primary caregivers’ education and occupation into a score ranging from 8 to 66, with higher scores indicating higher SES (Hollingshead, 2011). Twins and multiples were assigned to the same group. Families and clinical staff were not informed of group status.

### Procedures

#### Speech recordings

Following consent and prior to randomization, all mothers were audio-recorded while reading aloud the first chapter from the children’s storybook, Paddington Bear (Bond, 1958), in their native language. This text was selected because it is available in multiple languages, permitting consistency across participants, and because it captures a rich and continuous sample of maternal speech. To encourage infant-directed speech, mothers were instructed to read the text as though they were reading to their baby. Recordings were subsequently post-processed to have the same sound intensity (45 dB) and divided into two 10-min segments using the auditory software Praat.^1^

#### Delivery of intervention

The intervention began after infants transitioned from the NICU to an intermediate care nursery, a transition used as a general marker of increasing medical stability. Transition to the intermediate care nursery required that infants, regardless of study involvement, be on high-flow nasal canula (HFNC) at 2 liter or less and receiving enteral feeds delivered via nasogastric (ng) or by mouth (PO). Infants were considered ready for the intervention and other multimodal sensory interventions if they demonstrated cardiorespiratory stability consistent with criteria outlined in published unit protocols, the i-Rainbow Stage 5, Byrne et al. (2024) defined as tolerating routine care, intentional voice exposure, and skin-to-skin contact with recovery to baseline vital signs within approximately 15 min. We began the intervention at or after 32 weeks’ post-menstrual age (PMA), when the requirements above were met, to ensure adequate auditory maturation for the perception of speech sounds (Hall, 2000). As a result of these precautions, the participants had few major medical complications. Audio recordings of maternal speech were played for infants randomized to the T-group during the overnight hours of 10pm – 6am, a period of relative quiet in the intermediate care unit open bay setting. This timing helped to ensure that infants could perceive the recordings while also minimizing the risk of exceeding safe sound levels. Administration of the intervention overnight, during periods when parents were unlikely to be present at beside, was also expected to minimize parental knowledge for group assignment. Within each hour, two separate 10-min segments were played at pre-specified intervals (10:00 and 10:30pm, 11:10 and 11:40pm; 12:20 and 12:50pm; 1:00 and 1:30am; 2:10 and 2:40am, 3:20 and 3:50am, 4:00 and 4:30am; 5:10 and 5:40am). These staggered intervals were designed to minimize synchronization with infants’ biological rhythms or routine caregiving and feeding times. Thus, in a single night an infant in the T-group heard a total of 20 min/h for a total of 8 h [(10 min × 2) × 8 h = 160 min/night]. Infants in the T-group thus heard a total of approximately 2.67 h (160 total minutes/60 min) of speech recordings per night far above the average of 20–50 min, the usual speech exposure of infants cared for in open-bay NICU settings (Caskey et al., 2011). Recordings were played automatically via a timer function on an iPod touch (6th generation) to minimize involvement of clinical staff and to ensure regular timing of recordings. All iPods were placed in a shower caddy that was suctioned to the wall of the incubator/crib. This placement ensured iPods remained at a safe distance (>15 cm) from an infant’s head and allowed for continuous power to the iPod. Equipment was biosafety and infection control approved and sanitized using hospital-grade disinfecting wipes before crib placement and after removal. Sound intensity for speech recordings was played below hourly safety levels < 50 dB (5). The research team made regular visits at bedside to ensure proper device placement and functioning. To keep families and staff from determining group status, infants randomized to the C-group had their mothers record themselves reading and had the same the auditory setup; however, no recordings were played. Duration of intervention was defined as number of nights between when first nightly recording began or mock auditory set-up was first placed in a crib to the date of MRI scan, which typically preceded hospital discharge (i.e., approximately 36–38 weeks PMA). The percentage of days parents were charted as being present at the bedside during the intervention period was also measured to approximate balance in caregiver speech between groups.

#### Intervention fidelity and safety monitoring

Various procedures were implemented to ensure infant safety and to monitor infant well-being. Staff education included multiple in-person presentations and breakroom signage to inform physicians and nursing staff about the study. The research team attended weekly multidisciplinary clinical rounds to assess infant eligibility for study participation and health monitoring of enrolled participants. In addition, bedside signage in English and Spanish was provided for all participating infants to (1) indicate to clinical staff and families that a baby was enrolled in our study (2) provide telephone and email contact information for the PI and research staff (3) request that volume settings not be adjusted to ensure safe sound levels, although this possibility was unlikely given that devices were locked with a numeric code known only to study staff. In multiple instances, nurses used contact information to notify the research team when a baby was moving beds so that equipment could be transferred. These calls were made on weekdays and weekends at all hours. The research team did not receive reports from nursing staff about safety, health concerns, or sleep disruption via phone calls, email, or direct conversations during multidisciplinary rounds or when research staff visited the NICU to monitor device performance and recruit families for study participation. The research team also did not receive notification for any safety concerns during regular bedside visits performed regularly to ensure device placement, functioning and volume settings. Clinical variables including significant cardiorespiratory events [apneas, bradycardias, oxygen desaturation (i.e., ABD)] requiring nursing intervention, growth/weight gain, and overall length of NICU stay were also collected from routine charting to ensure that the intervention did not adversely impact infant health. All study equipment including iPods and charging equipment was thoroughly sanitized with hospital-grade wipes prior to placement in cribs and following any crib change to limit any risk of infection.

#### Clinical MRI scanning and sequence parameters

At the time of the study, standard of care at LPCH for all infants born very preterm (<32 weeks gestational age) included MRI scans at near-term-equivalent age, prior to hospital discharge. Per clinical procedures, infants were scanned during natural sleep without the use of sedation. For this study, the majority of infants were scanned on a 3-T MRI (GE-Discovery MR750) equipped with an 8-channel HD head coil (*n* = 28). Following upgrades to clinical equipment, five infants were scanned using a 32-channel (*n* = 5) HD head coil (General Electric Healthcare, Little Chalfont, UK). Scans were collected by hospital technologists unaware of group assignment. For purposes of this study, three additional MRI sequences were collected at the time of routine imaging. First, we collected a 60-direction (high-angular resolution) diffusion MRI sequence scan 1: *b* = 700 s/mm^2^ with multi-slice echo-planar imaging (MS-EPI) for rapid image acquisition (∼3 mins) and two-six volumes *b* = 0. Second, we collected a second additional 60-direction diffusion MRI sequence scan 2: *b* = 1500 s/mm^2^ was included in the clinical neuroimaging protocol as a back up scan (∼3 mins). It is used here to assess the reliability of findings from scan 1. Thirdly, we collected a quantitative T1 relaxometry sequence scan, performed with a slice-shuffled, inversion recovery EPI (IR-EPI) sequence with multiple inversion times (TI) and a second IR-EPI with the reverse-phase encoding direction (∼3 mins). The qT1 relaxometry sequence was used here as a reliability check of dMRI findings and to help interpret the potential neurobiological properties, given evidence that R1 signal (1/T1) is strongly correlated with myelin content in white matter regions (Stüber et al., 2014). Both dMRI and qT1 sequences were collected with a spatial resolution of 2.0 mm^3^ with full brain coverage. The clinical protocol included a high-resolution T1-weighted sequence (1 mm^3^ voxel size) that was used as the participant-specific anatomical reference for analyses of dMRI data. Total scan time was ∼30 min including dMRI and qT1 sequences.

#### Neuroimaging pre-processing and tractography analysis

Magnetic resonance imaging (MRI) data were managed and analyzed using a cloud-based neuroinformatics platform.^2^ Procedures our team developed for diffusion MRI pre-processing and tractography analysis of neonatal clinical MRI scans are described in Dubner et al. (2023). All procedures employed a neonatal template [Edinburgh Neonatal Atlas (ENA33)] for diffusion tractography analyses. Details for these procedures are provided in the supplemental information along with additional procedures for qT1 preprocessing and analyses (see Supplemental information). These procedures were used to obtain the primary outcome measure, mean diffusivity (MD) and secondary outcome measures, fractional anisotropy (FA) and relaxation rate (R1 sec^–1^) from the left and right arcuate fasciculus tracts.

### Outcomes

The primary outcome was white matter mean diffusivity (MD) of the left and right arcuate fasciculus. *Post hoc* secondary outcome measures included fractional anisotropy (FA) from dMRI and relaxation rate R1 (R1 = 1/T1) from quantitative T1 relaxometry scans also measured from the left and right arcuate fasciculus. We hypothesized that decreases in MD and increases in FA and/or R1in the treatment group, compared to the control group, would provide evidence of increased maturation within the arcuate fasciculus.

The present intervention was considered minimal risk. Study monitoring was assessed by the PI, the institutional review board and an internal data safety monitoring board comprised of study consultants, including board certified neonatologists and a pediatric neuroradiologist at Stanford Medical School. No adverse events were reported.

### Study ethics

The study protocol was approved by the Stanford School of Medicine Institutional Review Board (protocol #32638). The trial is listed as Listening to Mom in the NICU: Neural, Clinical and Language Outcomes # NCT02847689 on clinicaltrials.gov. Written informed consent was obtained from parents for all participants. No external data monitoring committee was used for this study.

### Statistical analysis

We planned enrollment of *n* = 42 participants (21 per group) based on power calculations that used data from an existing diffusion MRI intervention study of preterm infants born preterm (Als et al., 2004). Planned enrollment was expected to have power of ß = 0.94 to detect significant group differences of large effect size (Cohen’s *d* = 1.0). All statistical analyses were performed in R (version 4.3.1). We used an intention-to-treat strategy for all analyses. All major study variables were inspected for normality visually using histogram plots and statistically using Shapiro-Wilk normality tests. All primary and secondary outcome measures were normally distributed (all *p* > 0.05). Linear mixed models were used to assess group differences on the primary outcome measure, with treatment group as a fixed effects and family group included as a random effect to account for clustering of twins and multiples. Secondary analyses using linear mixed models were performed to confirm treatment effects in fractional anisotropy (FA) from dMRI scans and relaxation rate (R1) from the quantitative T1 relaxometry scan. The effect size of group differences was reported as a marginal R^2^. The residuals of all linear mixed-effects models were confirmed to be normally distributed based on histogram plots and Shapiro-Wilk normality tests (all ps > 0.05). Exploratory analyses used Pearson correlations to examine associations between the primary outcome metric (MD) and additional white matter metrics (FA, R1), in order to explore potential neurobiological properties related to intervention effects in arcuate tracts. We anticipated that if the underlying properties indexed by diffusion metrics (MD, FA) reflected tissue characteristics such as myelin content, we would observe negative correlations between MD and R1 and positive correlations between FA and R1. Additional exploratory analysis examined whether intervention effects generalized to other white matter tracts. These analyses were performed in pathways that traverse anterior or posterior segments of the corpus callosum (see Supplementary Material for procedures). We chose these tracts to capture white matter regions that are non-overlapping with the arcuate and that are generally not implicated in speech processes. Statistical significance was set at for all analyses *p* < 0.05.

### Role of the funding source

The funder of the study had no role in study design, data collection, data analysis, data interpretation or writing of the report.

## Results

**FIGURE 1:**
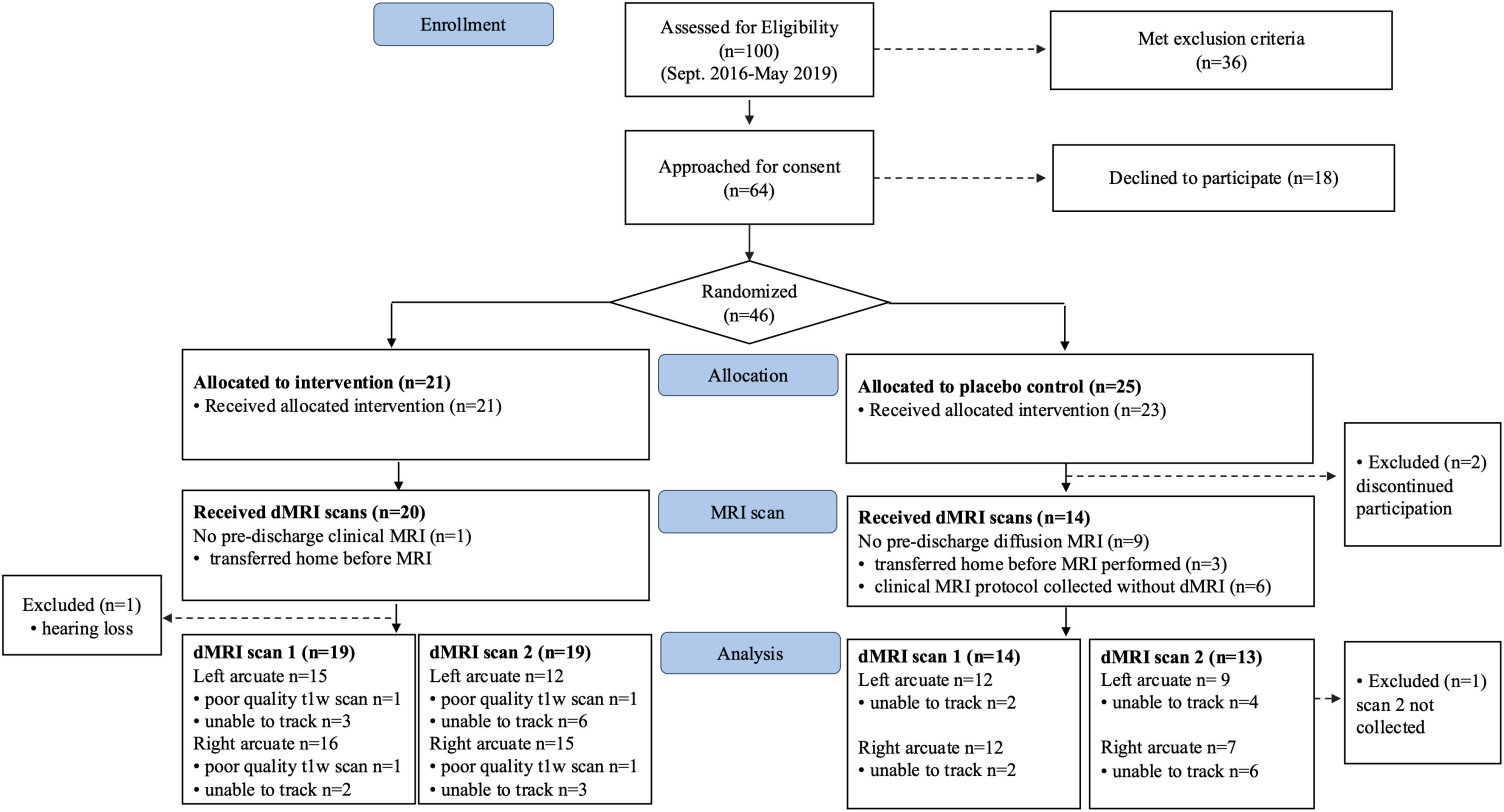
Consort diagram.

Between September 2016 and May 2019, a total of 46 neonates born very preterm were consented and randomized to either the treatment group (*n* = 21) or control (*n* = 25) groups (Figure 1). From the initial sample, 33 neonates (*n* = 19 T group; 14 C group) had diffusion MRI scans available for analysis. After attrition, the analyzed sample had power of ß = 0.8 to detect large effects (Cohen’s *d* ≥ 1.0). Baseline clinical, demographic and intervention characteristics were matched between groups in both the initial randomized sample (Table 1) and in the final sample with available dMRI data (*n* = 33) (Supplementary Table 1).

**TABLE 1:** Descriptive statistics [M(SD) or (*n*(%)] for baseline participant characteristics, intervention procedures, and safety monitoring data.

Participant characteristics	Treatment	Control	*t* or X^2^
Participant count	*n* = 20	*n* = 23	
GA at birth (weeks)	29.8 (2.3)	29.7 (2.0)	−0.02
Birthweight (g)	1362.5 (407.3)	1348.9 (346.0)	−0.12
SES^1^	47.8 (17.3)	45.9 (14.8)	−0.38
Male (%)	12 (60.0%)	11 (47.8%)	0.03
Mechanical ventilation (days)	1.4 (4.3)	2.1 (4.9)	0.52
Antenatal corticosteroids (%)	18 (90.0%)	20 (87.0%)	0.01
Apgar at 1 min	6.4 (2.4)	5.9 (2.3)	−0.62
Apgar at 5 min	8.0 (1.3)	8.1 (1.1)	0.36
White matter injury (%)^2^	1 (5.2%)	3 (15.0%)	0.82
**Intervention procedures**			
Voice recording in English (%)	16 (80.0%)	17 (73.9%)	0.22
PMA at start of intervention	33.6 (0.8)	34.0 (0.9)	1.33
Total days of intervention	15.3 (8.7)	14.7 (6.1)	−0.27
Parent presence at bedside during intervention^3^	68.2 (0.2)	72.0 (0.2)	0.65
Pre-discharge MRI collected (%)	19 (95.0%)	20 (87.0%)	0.82
PMA at MRI	36.0 (1.2)	36.0 (1.2)	0.03
**Safety monitoring**			
Apnea, bradycardia, desaturations (ABD) events per day during intervention^4^	0.38 (0.60)	0.51 (0.66)	0.66
Total ABD events during intervention^5^	7.95 (13.62)	9.83 (16.72)	0.41
Weight gain during intervention (g)	575.7 (390.9)	496.5 (217.5)	−0.80
Length of stay (days)	49.2 (21.7)	55.0 (25.8)	0.80

^1^SES as indexed by the Hollingshead Index;

^2^Mild punctate white matter injury as documented by pediatric neuroradiologists who interpreted clinical pre-discharge MRI. Percentage calculated out of infants with MRI scans;

^3^Number of days during the intervention that parents were charted as present at bedside/total days of intervention;

^4^Total number of significant apnea, bradycardia or desaturation events/total days of intervention;

^5^Total number of significant apnea, bradycardia or desaturation events.

Table 1 reports clinical safety monitoring metrics, revealing no significant differences between treatment and control groups in significant cardiorespiratory events, weight gain during the intervention period, or overall length of NICU stay.

**FIGURE 2:**
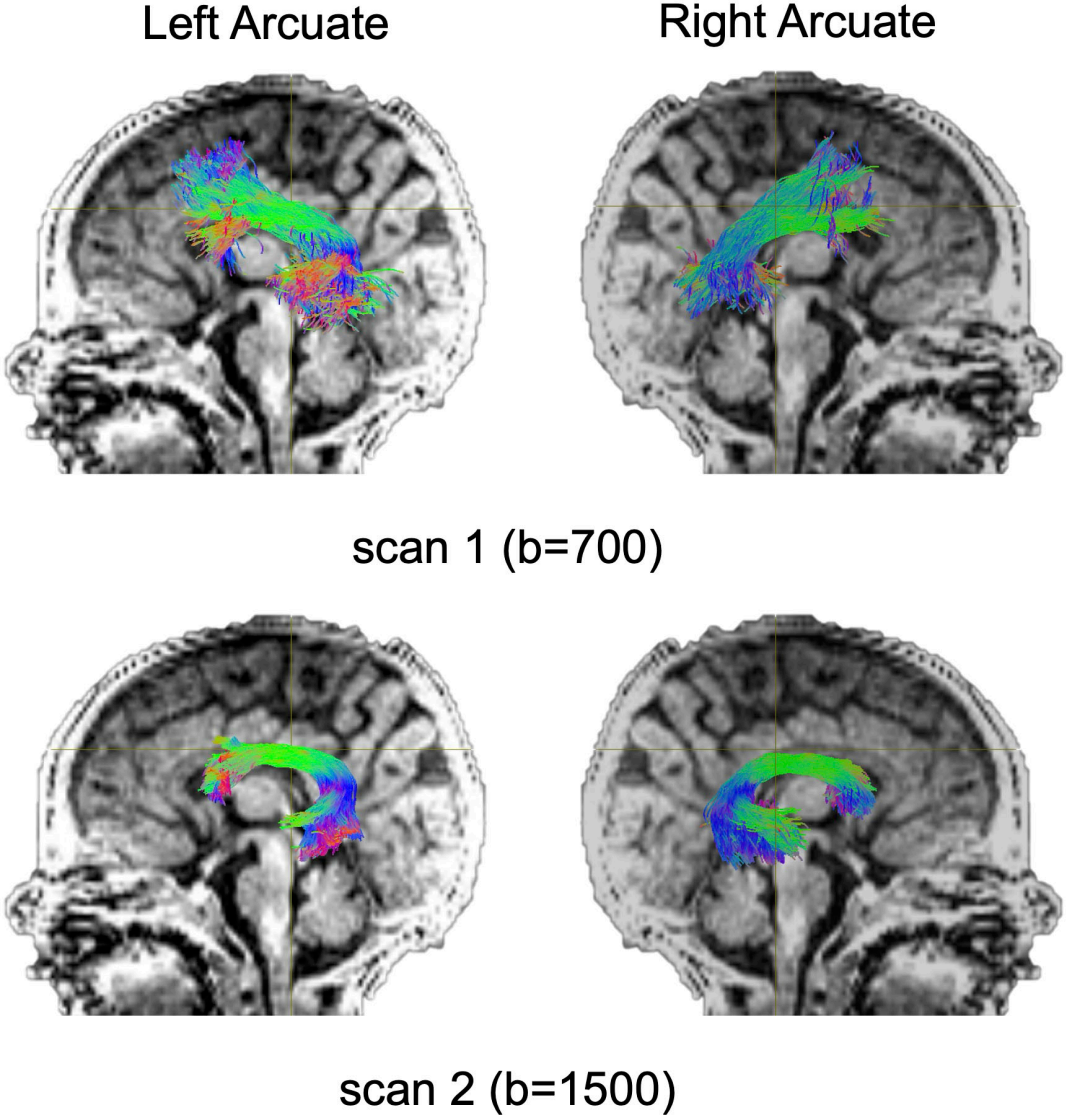
Tract renderings illustrate the left and right arcuate fasciculus from a single infant participant born very preterm. Tract renderings from each of two separate diffusion MRI scans (scan 1, b-value = 700 or b-value 1500) are displayed on a mid-sagittal T1-weighted (T1w) image. Colors represent primary orientation of streamlines (red, left-right; green, anterior-posterior; blue, superior-inferior).

**FIGURE 3:**
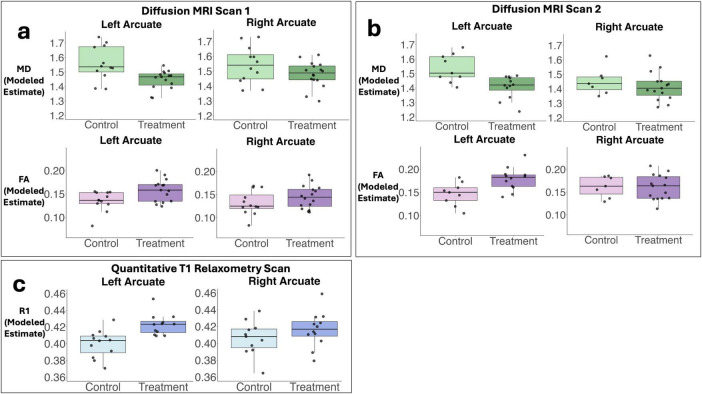
Box plots demonstrate predicted values for primary and secondary outcome measures used to assess white matter microstructure. Panel (a) demonstrates modeled estimates (adjusting for family clustering as a random effect) for mean diffusivity (MD, primary outcome) and fractional anisotropy (FA, secondary outcome) obtained from the left and right arcuate from diffusion scan 1 (b-value = 700). Panel (b) shows modeled group estimates for MD and FA obtained from the left and right arcuate from diffusion scan 2 (b-value = 1500). Panel (c) illustrates modeled group estimates for the secondary outcome measure relaxation rate (R1) obtained from the left and right arcuate from the quantitative T1 relaxometry scan. Box plots represent adjusted medians and quartiles. Gray scatter dots represent individual raw data points for participants.

Diffusion MRI tractography analyses was successful in identifying the left and right arcuate in the majority of infants for both scans (scan 1: left 82%; right 85%; scan 2: left 66%; right 69%). Figure 2 shows tractograms of the left and right arcuate fasciculus generated from each of the two dMRI scans (b-value = 700 and b-value = 1500) and displayed on the T1w image from a single participant. Table 2 presents group means for primary (MD) and secondary (FA, R1) outcome measures and results of statistical analyses performed to assess group differences. Figure 3 presents box plots to visualize group comparisons for MD and FA measured from both dMRI scans (scan 1 and scan 2) and R1 measured from the qT1 scan. *Post hoc* analyses confirmed that infants with successful tracking in the arcuate were not statistically different (*p* > 0.05) from infants without tracking or diffusion MRI scans on any clinical or demographic factor.

**TABLE 2:** Results of linear mixed-effects models for group comparisons of white matter microstructure metrics.

Outcome measures	Coefficient (SE)	Treatment estimated mean (95% CI)	Control estimated mean (95% CI)	*t*	Marginal R^2^
**Primary outcome: mean diffusivity (MD)^1^**					
**Scan 1**					
Left arcuate fasciculus	−0.11 (0.04)	1.45 (1.40–1.50)	1.56 (1.49–1.62)	−3.11**	0.27
Right arcuate fasciculus	−0.06 (−0.03)	1.48 (1.42–1.53)	1.54 (1.47–1.61)	−1.58	0.09
**Scan 2**					
Left arcuate fasciculus	−0.12 (−0.04)	1.41 (1.36–1.46)	1.53 (1.46–1.59)	−3.04**	0.33
Right arcuate fasciculus	−0.03 (−0.04)	1.42 (1.36–1.47)	1.44 (1.36–1.53)	−0.6	0.01
**Secondary outcome: fractional anisotropy (FA)^2^**					
**Scan 1**					
Left arcuate fasciculus	0.02 (−0.01)	0.16 (0.15–0.17)	0.14 (0.12–0.15)	2.58*	0.20
Right arcuate fasciculus	0.01 (0.01)	0.15 (0.13–0.16)	0.13 (0.11–0.15)	1.35	0.06
**Scan 2**					
Left arcuate fasciculus	0.03 (0.01)	0.18 (0.16–0.19)	0.15 (0.13–0.17)	2.96**	0.31
Right arcuate fasciculus	−0.002 (0.01)	0.16 (0.15–0.17)	0.16 (0.13–0.19)	−0.14	0.001
**Secondary outcome: relaxation rate (R1)^3^**					
**Scan 3**					
Left arcuate fasciculus	0.02 (0.01)	0.42 (0.41–0.43)	0.40 (0.39–0.41)	3.83***	0.39
Right arcuate fasciculus	0.01 (0.01)	0.42 (0.40–0.43)	0.41 (0.40–0.42)	0.84	0.03

^1^MD, mean diffusivity (μ^2^/ms);

^2^FA, fractional anisotropy scalar value 0-1;

^3^R1, relaxation rate (1/s);

*p < 0.05;

**p < 0.01;

***p < 0.001.

Analysis of MD, the primary outcome measure, obtained from the arcuate tracts revealed that neonates in the T-group demonstrated significantly lower MD compared to neonates in the C-group in the left but not the right arcuate in both dMRI scans (Figure 3 and Table 2). Effect sizes in the left arcuate were large, (explaining 27%–33% of the variance in MD) while effect sizes in the right arcuate were small (1%–9% of the variance in MD). Secondary *post hoc* analyses of FA from dMRI scans and R1 from the quantitative T1 relaxometry scan confirmed treatment effects within the left arcuate fasciculus, with neonates in the treatment group demonstrating significantly higher FA and higher R1 compared to neonates in the control group (Figure 3 and Table 2). Effect sizes in the left arcuate were medium to large, explaining 20%–31% of the variance in FA and 39% of the variance in R1. No effect of treatment was observed within the right arcuate for either FA or R1, and effect sizes were small (explaining 1%–6% of the variance in FA and R1) (Figure 3 and Table 2). *Post hoc* sensitivity analyses confirmed that the pattern of results for all white matter metrics were the same and remained significant including head coil as a covariate or after excluding three infants (2 controls, 1 treatment) with evidence of mild punctate white matter injury.

Exploratory analyses performed to examine potential neurobiological properties impacted by the intervention revealed that all metrics of white matter microstructure were associated in expected directions. Specifically, MD was observed to be strongly and negatively correlated with both R1 and FA in the left and right arcuate, while FA and R1 showed moderate positive correlations in the left and right arcuate (Supplementary Figure 1).

Exploratory analysis performed in the anterior and posterior corpus callosum revealed no significant differences between the T-group and the C-group in either segment for any outcome measure (Supplementary Table 2). Effect sizes were small to medium (explaining 3%–13% of the variance in white matter metrics).

## Discussion

This randomized control trial established the effect of increased recorded speech exposure during NICU hospitalization on structural brain development in a relatively healthy sample infants born very preterm. Our findings indicated that the intervention was safe and did not negatively affect infants’ cardiorespiratory stability, growth, or length of hospital stay. Compared to infants in the control group, infants who experienced increased exposure to maternal speech demonstrated evidence for more mature microstructure of the left arcuate fasciculus. The left arcuate is presumed critical for language given that is forms structural connections between inferior parietal/posterior temporal cortices, regions implicated in speech-sound recognition, to inferior frontal cortices, regions implicated in motor-speech processes (Catani et al., 2005; Ivanova et al., 2021; Poeppel and Hickok, 2004; Poeppel et al., 2016; Saur et al., 2008). Importantly, group differences were robust, replicating across three separate MRI scans (two diffusion sequences and quantitative T1 relaxometry) and three measures of white matter microstructure (MD, FA and R1). These findings go beyond previous correlational studies to causally link speech experiences during neonatal development to the maturation of brain structures implicated in language. Moreover, these findings suggest that enriching the speech environment of the NICU may facilitate the healthy maturation of white matter pathways, offering important benefits to structural brain development in children born preterm.

Consistent with our hypotheses, neonates in the treatment group demonstrated significantly lower MD compared to neonates in the control group. This finding aligns with previous studies showing reductions in MD and/or increases in FA in response to language-based interventions in children (Huber et al., 2018; Keller and Just, 2009), or in response to parent-based NICU interventions for preterm newborns (Als et al., 2004). Exploratory *post hoc* analyses of our data found strong negative correlations between our primary outcome measure, MD, and R1, a metric from qT1 relaxometry, that has been shown to be highly predictive of myelin content in histological studies (Stüber et al., 2014). Increases in myelin content and reductions in water content from tissue growth are characteristic of changes occurring prenatally during the third trimester and postnatally through the 2nd year of life (Kinney et al., 1988; Yakovlev and Lecours, 1967). These developmental changes in tissue properties are detectable with the white matter microstructural metrics assessed here (MD, FA, and R1) (Gelman et al., 2001; Stüber et al., 2014) and are presumed to reflect myelination (Yeatman et al., 2014). Thus, our findings provide evidence that the intervention led to differences in tissue properties that reflect enhanced white matter maturation.

The large effect size in the left arcuate is notable. Effect sizes of similar magnitudes have been reported for dMRI metrics, such as MD and FA, comparing term to very preterm infants at term-equivalent ages (Dibble et al., 2021). The amount of additional speech provided in the treatment condition was comparable to the difference between maternal speech exposure *in utero* during the third trimester and that experienced by preterm neonates of equivalent ages ex-utero (Monson et al., 2023). Our ability to detect large effects may thus be because the current intervention closely approximated daily levels of speech exposure necessary for brain development. Further randomized trials are needed to determine the specific dosing and timing of speech exposure needed to remediate gaps in speech exposure that can occur during NICU hospitalization and that may adversely impact brain development (Rand and Lahav, 2014).

Treatment effects were largest in the left arcuate, with small effects in the right arcuate and minimal effects in anterior and posterior segments of the corpus callosum. The current study was powered to detect large effects. Subsequent studies with larger samples are needed to directly test whether intervention effects are specific to language pathways across hemispheres or may generalize to additional white matter pathways. Differences in white matter properties of anterior and posterior segments of the corpus callosum have been observed in multiple studies comparing term and preterm neonates and children (Anjari et al., 2007; Li et al., 2015; Travis et al., 2019) that may relate to whether a preterm child experienced neonatal inflammatory conditions (Anderson et al., 2024; Dubner et al., 2019). Larger studies are also needed to establish the efficacy of our intervention for children born preterm with more complex medical histories who were not included here but may be at most risk for alterations in white matter development from long hospital stays, inflammatory conditions, and other complications of preterm birth.

Most evidence to date linking early speech experiences to structural brain development derives from observational studies in older infants (Fibla et al., 2023). The current findings provide causal evidence of a direct effect of speech experiences on neonatal brain development. Previous randomized controlled trials of premature newborns have demonstrated effects of non-speech auditory stimuli, such as biological maternal sounds or non-vocal music therapy, on structural and functional neonatal brain development, within gray and white matter areas implicated in processing auditory and socio-emotional information (Lordier et al., 2019; Sa de Almeida et al., 2023; Webb et al., 2015). Our work extends these findings by demonstrating that auditory experiences directly related to maternal speech influence the development of a key brain structure, the left arcuate, which has been implicated speech perception and production processes. Together with these previous studies, the current findings underscore the sensitivity of the neonatal brain to auditory-speech experiences.

Understanding whether different types of auditory and speech experiences have similar, differential, or combinatorial benefits on neonatal structural brain development requires further investigation. For example, future NICU interventions could test whether infant- versus adult-directed registers have similar or differential effects on structural brain development. Behavioral and functional neuroimaging studies using EEG in preterm newborns have shown preferences for and perceptional sensitivity to infant-directed speech (Butler et al., 2014; Kjeldsen et al., 2025; Neel et al., 2023), which has established benefits for language learning and outcomes in older infants and toddlers (Kuhl, 2004). In contrast, speech overheard in the womb is likely to involve spontaneous conversations rich in adult-directed registers. Both forms of input may support auditory-speech processing abilities, such as phoneme perception, which likely depend on pathways within the arcuate fasciculus. However, because adult-directed speech typically provides greater lexical diversity, while infant-directed speech emphasizes prosodic and affective cues, these two forms of input may also engage different and additional brain networks. Adult-directed speech may preferentially engage left superior and anterior temporal areas, whereas infant-directed speech may more strongly recruit homologous right-hemisphere regions (Poeppel et al., 2016). Directly contrasting these input types within a single study will be essential for clarifying their distinct contributions to brain development and for optimizing the design and delivery of speech-based interventions in the NICU.

Auditory therapies and speech interventions may be effective in promoting brain development in preterm newborns possibly by reducing stress or by providing experience-dependent activity to promote neuronal functioning (Anderson and Patel, 2018; Rand and Lahav, 2014). Recorded maternal speech in the NICU may also benefit sleep in preterm newborns, a factor relevant for brain development and which may have also been affected by the current intervention (Shellhaas et al., 2019). Relating measures of structural brain development to physiological or clinical assessments of neurological and neurobehavioral functioning will be important for interrogating the neural mechanisms of early NICU auditory interventions. Studies able to measure neurobehavior and physiological responses are also needed to understand the contributions of NICU speech interventions to functional outcomes. Identifying the underlying neurobiological correlates of such interventions will also benefit from translational research studies in non-human animals.

Our findings indicated that the current intervention was safe for medically stable preterm infants with minimal health complications. Further studies are needed to assess the safety and efficacy of similar recorded speech interventions in infants with more severe health conditions. Previous research has aligned auditory interventions with infant state regulation (Sa de Almeida et al., 2023), a consideration that especially crucial for medically fragile infants or those with severe illness. Newly developed tools for monitoring infant sleep states (de Groot et al., 2022) could play a key role in optimizing the delivery of speech interventions and other developmental care practices to enhance neuroprotection in support of neurodevelopment.

The current study highlights how clinical interventions can serve a dual purpose, advancing theoretical understanding about how environmental experiences directly contribute to structural brain development, while simultaneously identifying strategies to promote positive outcomes for children born preterm. In addition, the implementation of such interventions may foster new opportunities to educate parents about the importance of talking and reading to their babies, both during and beyond the NICU hospitalization. Importantly, this work also has implications for addressing healthcare inequities. Research has shown that developmental care practices in the NICU can vary by family socioeconomic status (Brignoni-Pérez et al., 2021); families of lower-SES face barriers such as limited paid leave, transportation costs, and competing childcare needs, which can limit their ability to be at bedside. Providing recorded caregiver speech could thus help to reduce these disparities by ensuring that all infants receive at least minimal exposure to parental speech, supporting their behavioral and brain development.

This study has limitations. The sample size was small, although adequately powered to detect large effects. Our replication of treatment effects across three separate scans and across three measures provides confidence in our conclusions. We did not have baseline scans to establish whether differences in neural structures predated intervention. This concern is minimized because randomization resulted in groups with comparable medical and demographic profiles in factors that could influence neonatal brain development, such as gestational age and postmenstrual age at scan. This balance remained after accounting for attrition in participants with available scans, another limitation of the present study. While we did not have a direct measure of caregiver speech at bedside, parental presence in the NICU was comparable between groups and similar to published rates at our center (Lazarus et al., 2024). Parents or nursing staff may have learned of group assignments and altered their behavior. However, several factors make this unlikely to have occurred or to have significantly biased the results. First, procedures minimized the chance of group status being revealed (e.g., all mothers recorded speech prior to randomization, all infants had an iPod in their crib regardless of group assignment, and recordings were played overnight between the hours of 10pm–6am when families were typically absent). Second, the similarity in parental presence in the NICU between groups suggests that parental behavior did not differ significantly as a function group status. Third, nursing staff rotated shifts every 2–3 days, were not involved in MRI data analysis or collection, and were unlikely to have capacity in their schedule to modify behaviors for individual babies dependent on their involvement in the intervention study and/or group assignment. This cohort was not representative of the full range of infants born very preterm, as our sample was limited to relatively healthy preterms. At the same time, this design feature is also a strength because our findings may more easily generalize to models of brain development in typical developmental circumstances. Larger samples are needed to replicate these findings and to determine the generalizability of intervention effects across infant of varying gestational ages. Additional studies are also important for testing potential dose-response intervention effects and potential long-term effects of the intervention on language outcomes.

In sum, increasing exposure to maternal speech via overnight audio recordings was directly linked to maturation of the left arcuate fasciculus, a white matter circuit implicated in life-long language processing. The study provided a scientifically necessary causal link between language experience and brain structure. These findings also offer key insights regarding how interventions involving increasing exposure to speech may offer important neuroprotective experiences that can mitigate the effects of prematurity on brain development.

## Data Availability

The datasets presented in this article are not readily available because of patient privacy issues but de-identified data are available from the corresponding author on reasonable request and subject to institutional approvals.

## Footnotes

1
http://www.fon.hum.uva.nl/praat/

2
https://flywheel.io/
