# Supplementary material for: Listening to mom in the neonatal intensive care unit: a randomized trial of increased maternal speech exposure on white matter connectivity in infants born preterm
Source: Front Hum Neurosci. 2025 Oct 14;19:1673471. doi: 10.3389/fnhum.2025.1673471 (PMC12558918; doi:10.3389/fnhum.2025.1673471)
Supplement: Supplementary file 1 [file Data_Sheet_1.pdf]

Supplemental Information

Image Preprocessing

Diffusion MRI

Analyses of neuroimaging dMRI data was implemented in Reproducible Tract Profiles (RTP) (https://github.com/vistalab/RTP-pipeline) [1,2]. Diffusion preprocessing, modeling and tractography within RTP is largely based on open-source software from FSL (https://fsl.fmrib.ox.ac.uk/fsl/fslwiki), MRTrix3 (https://www.mrtrix.org/) and Automated Fiber Quantification (AFQ, https://github.com/yeatmanlab/AFQ) [3]. A full list of software dependencies can be found here (https://github.com/vistalab/RTP-pipeline/wiki). Procedures for preprocessing were the same for the two diffusion scans collected at different b-values (b=700 or b=1500). RTP consists of three main steps: (i) Structural processing and Region of Interest (ROI) creation (ii) dMRI preprocessing (RTP-preproc) and (iii) whole-brain tractography and tract segmentation (RTP-Pipeline). Details of these steps are described by Lerma-Usabiaga et al.[2] and Liu et al. [1]. We modified the first step of the RTP pipeline to account for the immature neonatal brain. These adaptations included using infant Freesurfer (https://surfer.nmr.mgh.harvard.edu/fswiki/infantFS) [4] for T1-weighted image segmentation and replacing the adult MNI template and ROIs with a neonatal template from the Edinburgh Neonatal Atlas (ENA33) [5] and corresponding neonatal ROIs to enhance ROI placement and tract identification. The brainmask generated by infant freesurfer for each participant was inspected, and in some cases, manually edited using mrTrix to ensure full-brain coverage.

We performed the following diffusion image preprocessing steps (RTP-preproc): (i) data denoising using principal component analysis [6,7], ii) Gibbs ringing correction [8], (iii) eddy current

and motion correction [9], and (iv) anatomical alignment of diffusion data to the average non-diffusion-weighted volumes, which were registered to the infant's high-resolution, ac-pc aligned T1-weighted anatomical image using rigid body transformation.

Quantitative T1 relaxometry

We used IR-EPI data to estimate relaxation rate R1 (R1 = 1/T1) in each voxel. This was achieved using t1fit (https://github.com/cni/t1fit) software implement in Flywheel. This software includes routines to (1) unshuffle slices within a volume of differing inversion times from slice-shuffled pulse sequence (2) perform epi-distortion correction using FSL topup tool [10,11] and (3) perform T1 fitting using algorithms based on those described in Barral et al. (2010) [12]. Output of the t1fit software is the estimated T1 in each voxel. qT1 maps were then aligned to dMRI b=0 maps from scan 1 (bvalue=700), using rigid-body alignment performed using ANTs [13] implement in Flywheel. qT1 maps were visually inspected for alignment and included as inputs to RTP-Pipeline to obtain T1 values for the left and right arcuate. We calculated (R1 = 1/T1) for each tract using mean T1 generate for each tract. We chose to report R1 for ease of interpretation given that higher values of R1 are associated with higher levels of tissue properties related to myelin [14].

Tractography Analyses

The preprocessed dMRI data output from RTP-preproc served as the input for diffusion metrics modeling, whole-brain tractography, and tract segmentation in RTP-pipeline. Aligned qT1 maps were supplied as inputs implemented in RTP2 [15]. White matter diffusion metrics (FA and MD) were calculated based on the diffusion tensor model. Constrained spherical deconvolution model (CSD) [16] with eight spherical harmonics (lmax = 8) was used to calculate fiber orientation distributions (FOD) for each voxel. To account for the immaturity of the neonatal brain, the FA

mask threshold was set to 0.15, and the FOD threshold to 0.08 for CSD-based tractography. The CSD- based tractography consisted of (i) Ensemble Tractography [17] to estimate the whole-brain white matter connectome. MRtrix3 generated three candidate connectomes with varying angle parameters and lengths (1: angle 45°, lengths 100, 50; 2: angle 25°, lengths 10, 50; angle 5° lengths 100,50)[18]. For each candidate connectome, a probabilistic tracking algorithm (iFOD2) was used with a step size of 1 mm, a minimum length of 10 mm. The three candidate connectomes were then concatenated into a single ensemble connectome (ii) Spherical-deconvolution Informed Filtering of Tractograms (SIFT) to improve the quantitative accuracy of the ensemble connectome by filtering out streamlines that do not align with the FODs, ensuring that streamline densities match the FOD lobe integral. The resulting ensemble connectome retained 500,000 streamlines. (iii) AFQ [3] to segment and refine the left and right arcuate fasciculus from the resulting ensemble connectome of each neonate. Mean tract mean diffusivity (MD), fractional anisotropy (FA), and relaxation rate (R1) were calculated for the core of the tract, defined by the same ROIs used for tract segmentation of the left and right arcuate fasciculi. These same tractography procedures were also used to obtain measures from white matter tracts that traverse the anterior and posterior segments of the corpus callosum that are visualized in Supplemental Figure S2. Diffusion MRI tractography was successful in identifying the anterior and posterior segments of the corpus callosum in the majority of infants for both scans (scan 1: anterior 82%; posterior 76%; scan 2: anterior 82%; posterior 58%).

Supplemental Tables

Supplemental Table S1. Trial intervention participant characteristics and intervention descriptives for the final sample with available diffusion MRI data.

	Treatment	Control	t or X^2
**Participant characteristics**	*n* = 19	*n* = 14	
GA at birth (wk)	29.7 (2.3)	29.8 (2.1)	0.22
Birthweight (g)	1364.2 (418.3)	1389.4 (382.1)	0.18
SES[1]	47.2 (17.5)	45.3 (15.2)	-0.32
Male (%)	11 (57.79%)	6 (42.9%)	0.73
Mechanical ventilation (days)	1.4 (4.3)	1.8 (4.3)	0.24
Antenatal corticosteroids (%)	17 (89.5)	13 (92.9)	0.11
Apgar at 1 min	6.3 (2.4)	5.4 (2.3)	-1.05
Apgar at 5 min	8.0 (1.4)	8.0 (1.1)	0.0
White matter injury (%)[2]	1 (5.2%)	2 (14.2%)	0.65
**Intervention descriptives**			
Voice recording in English (%)	15 (78.9)	12 (85.7)	0.25
PMA at start of intervention	33.6 (0.8)	34.0 (0.9)	0.66
Total days of intervention	15.8 (8.7)	16.2 (7.2)	0.15
Parent presence at bedside during intervention[3]	70.4 (0.2)	73.1 (0.2)	0.41
PMA at MRI	36.1 (1.3)	36.0 (1.2)	0.28
**Safety Monitoring**			
ABD events per day during intervention[4]	0.38 (0.60)	0.55 (0.79)	0.63
Total ABD events during intervention[5]	8.26 (13.92)	12.00 (20.68)	0.59
Weight gain during intervention (g)	597.53 (388.87)	546.07 (255.56)	-0.46
Length of stay (days)	50.69 (21.25)	58.65 (29.72)	0.85

[1]SES as indexed by the Hollingshead Index; [2] Mild punctate white matter injury as documented by pediatric neuroradiologists who interpreted clinical pre-discharge MRI; [3]Number of days during the intervention that parents were charted as present at bedside/total days of intervention; [4]Total number of significant apnea, bradycardia or desaturation events/total days of intervention; [5]Total number of significant apnea, bradycardia or desaturation events. < .05*; p < .01**; p < .001***

Supplemental Table S2. Pearson correlations between primary (MD) and secondary (FA, R1) outcomes of white matter microstructure.

	Diffusion Scan 1 *Pearson's r* (95% CI)		Diffusion Scan 2 *Pearson's r* (95% CI)	
	Left Arcuate	Right Arcuate	Left Arcuate	Right Arcuate
MD-FA	-0.52<br>(-0.75 - -0.18)	-0.75<br>(-0.88 - -0.53)	-0.87<br>(-0.94 - -0.69)	-0.68<br>(-0.86 - -0.36)
MD-R1	-0.82<br>(-0.92- -0.63)	-0.85<br>(-0.93 – -0.67)	-0.78<br>(-0.91 - -0.51)	-0.74<br>(-0.89 - -0.43)
FA-R1	0.64<br>(0.32 - 0.83)	0.79<br>(0.57 - 0.91)	0.69<br>(0.35 - 0.87)	0.56<br>(0.14 - 0.80)

MD= mean diffusivity; FA = fractional anisotropy, CI = confidence interval

Supplemental Table S3. Results of Linear Mixed-Effects Models for Group Comparisons of White Matter Microstructure in Anterior and Posterior Segments of the Corpus Callosum.

	Coefficient (SE)	Treatment Estimated Mean (95% CI)	Control Estimated Mean (95% CI)	*t*	Marginal R^2
**Primary outcome: MD**					
**Scan 1**					
Anterior Corpus Collosum	-0.04 (0.04)	1.56 (1.51-1.62)	1.60 (1.53-1.68)	-0.94	0.04
Posterior Corpus Collosum	-0.03 (0.04)	1.50 (1.45-1.55)	1.53 (1.47-1.60)	-0.85	0.03
**Scan 2**					
Anterior Corpus Collosum	0.02 (0.04)	1.50 (1.46-1.55)	1.48 (1.42-1.54)	0.65	0.02
Posterior Corpus Collosum	-0.02 (0.04)	1.41 (1.36-1.46)	1.43 (1.35-1.51)	-0.51	0.01
**Secondary outcome: FA**					
**Scan 1**					
Anterior Corpus Collosum	-0.003 (0.01)	0.18 (0.17-0.19)	0.18 (0.17-0.20)	-0.25	0.003
Posterior Corpus Collosum	-0.04 (0.02)	0.19 (0.16-0.23)	0.23 (0.19-0.27)	-1.53	0.09
**Scan 2**					
Anterior Corpus Collosum	0.03 (0.01)	0.21 (0.19-0.23)	0.18 (0.15-0.22)	1.7	0.13
Posterior Corpus Collosum	-0.002 (0.02)	0.26 (0.23-030)	0.26 (0.21-0.31)	-0.09	0.00
**Secondary outcome: R1**					
**Scan 3**					
Anterior Corpus Collosum	-0.001 (0.01)	0.40 (0.38-0.41)	0.40 (0.38-0.41)	-0.13	0.001
Posterior Corpus Collosum	0.010 (0.01)	0.41 (0.40-0.42	0.40 (0.38-0.41)	0.76	0.03

$p < .05$*; $p < .01$**; $p < .001$***

Supplemental Figure S1

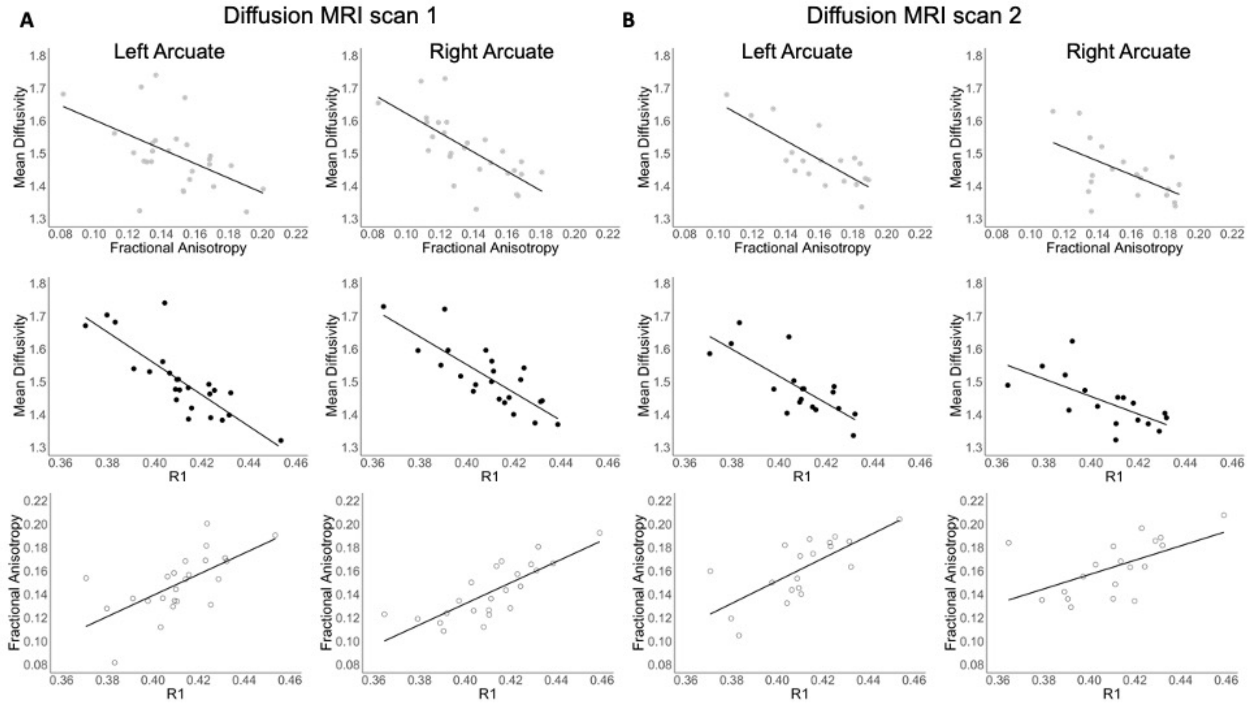

Supplemental Figure S1. Scatter plots depicting associations between primary (MD) and secondary outcome measures (FA, R1) of white matter microstructure. Panel A corresponds to diffusion MRI metrics MD and FA obtained from diffusion MRI scan 1 (bvalue = 700) and R1 obtained from the quantitative T1 relaxometry scan. Panel B corresponds to diffusion MRI metrics MD and FA obtained from diffusion MRI scan 2 (bvalue = 1500) and R1 obtained from the quantitative T1 relaxometry scan.

Supplemental Figure S2.

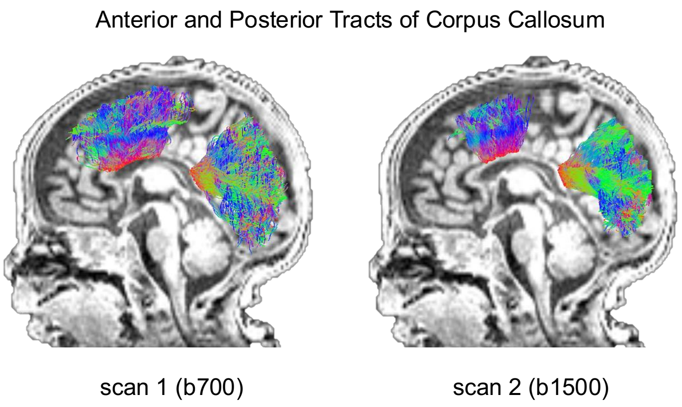

Figure S2. Tract renderings illustrate the white matter tracts that traverse anterior and posterior segments of the corpus callosum from a single infant participant born very preterm. Tract renderings from each of two separate diffusion MRI scans (scan 1, bvalue = 700 or bvalue 1500) are displayed on a mid-sagittal T1-weighted (T1w) image. Colors represent primary orientation of streamlines (red = left-right, green = anterior-posterior, blue = superior-inferior).

Supplemental Figure S3.

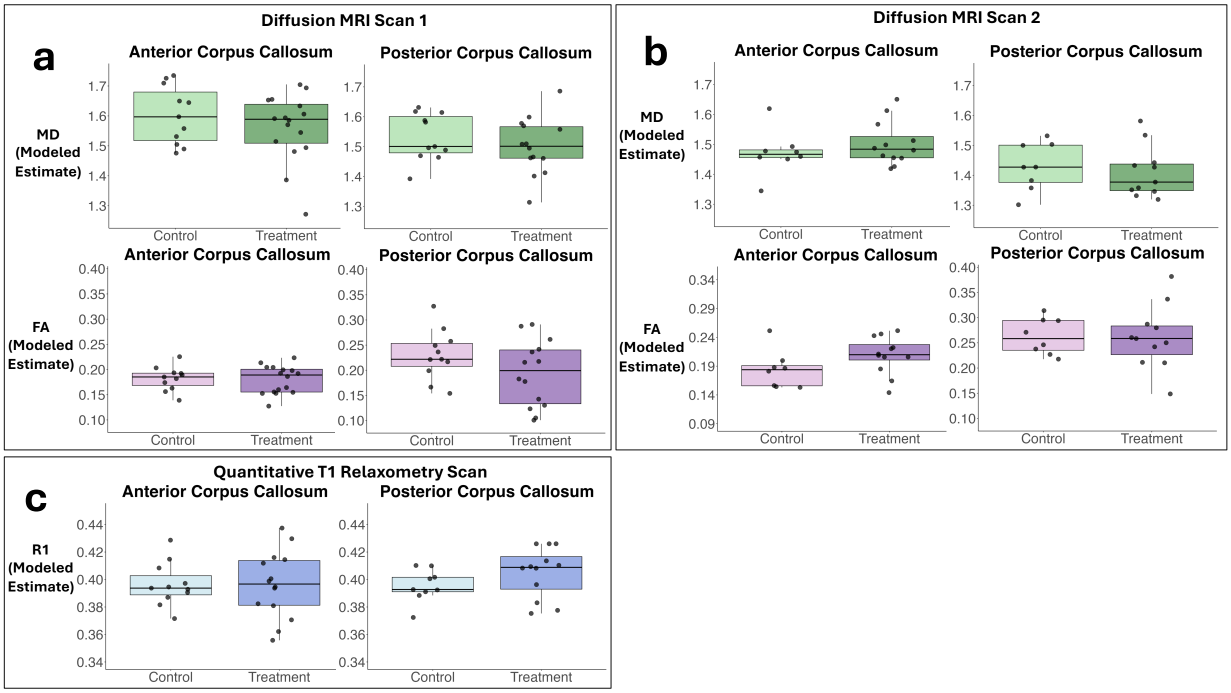

Supplemental Figure S3. Box plots demonstrate predicted values for primary and secondary outcome measures used to assess white matter microstructure in additional white matter tracts. Panel A demonstrates modeled estimates (adjusting for family clustering as a random effect) for mean diffusivity (MD, primary outcome) and fractional anisotropy (FA, secondary outcome) obtained from anterior and posterior segments of the corpus callosum from diffusion scan 1 (b-value = 700). Panel B shows modeled group estimates for MD and FA obtained from anterior and posterior segments of the corpus callosum from diffusion scan 2 (b-value = 1500). Panel C illustrates modeled group estimates for the secondary outcome measure relaxation rate (R1) obtained from anterior and posterior segments of the corpus callosum from the quantitative T1 relaxometry scan. Box plots represent adjusted medians and quartiles. Gray scatter dots represent individual raw data points for participants.

References

1. Liu, M., Lerma-Usabiaga, G., Clascá, F. & Paz-Alonso, P. M. Reproducible protocol to obtain and measure first-order relay human thalamic white-matter tracts. *NeuroImage* **262**, 119558 (2022).
2. Lerma-Usabiaga, G., Mukherjee, P., Perry, M. L. & Wandell, B. A. Data-science ready, multisite, human diffusion MRI white-matter-tract statistics. *Sci. Data* **7**, 422 (2020).
3. Yeatman, J. D., Dougherty, R. F., Myall, N. J., Wandell, B. A. & Feldman, H. M. Tract Profiles of White Matter Properties: Automating Fiber-Tract Quantification. *PLoS ONE* **7**, e49790 (2012).
4. Zöllei, L., Iglesias, J. E., Ou, Y., Grant, P. E. & Fischl, B. Infant FreeSurfer: An automated segmentation and surface extraction pipeline for T1-weighted neuroimaging data of infants 0-2 years. *NeuroImage* **218**, 116946 (2020).
5. Blesa, M. *et al.* Parcellation of the Healthy Neonatal Brain into 107 Regions Using Atlas Propagation through Intermediate Time Points in Childhood. *Front. Neurosci.* **10**, 220 (2016).
6. Veraart, J., Fieremans, E. & Novikov, D. S. Diffusion MRI noise mapping using random matrix theory. *Magn. Reson. Med.* **76**, 1582–1593 (2016).
7. Veraart, J. *et al.* Denoising of diffusion MRI using random matrix theory. *NeuroImage* **142**, 394–406 (2016).
8. Kellner, E., Dhital, B., Kiselev, V. G. & Reisert, M. Gibbs-ringing artifact removal based on local subvoxel-shifts. *Magn. Reson. Med.* **76**, 1574–1581 (2016).

9. Andersson, J. L. R. & Sotiropoulos, S. N. An integrated approach to correction for off-resonance effects and subject movement in diffusion MR imaging. *NeuroImage* **125**, 1063–1078 (2016).
10. Andersson, J. L. R., Skare, S. & Ashburner, J. How to correct susceptibility distortions in spin-echo echo-planar images: Application to diffusion tensor imaging. *NeuroImage* **20**, 870–888 (2003).
11. Smith, S. M. *et al.* Advances in functional and structural MR image analysis and implementation as FSL. *NeuroImage* **23 Suppl 1**, S208-19 (2004).
12. Barral, J. K. *et al.* A robust methodology for in vivo T1 mapping. *Magn. Reson. Med.* (2010) doi:10.1002/mrm.22497.
13. Avants, B. B., Tustison, N. & Song, G. Advanced Normalization Tools (ANTS). *Insight J.* (2011).
14. Stüber, C. *et al.* Myelin and iron concentration in the human brain: a quantitative study of MRI contrast. *NeuroImage* **93 Pt 1**, 95–106 (2014).
15. Lerma-Usabiaga, G., Liu, M., Paz-Alonso, P. M. & Wandell, B. A. Reproducible Tract Profiles 2 (RTP2) suite, from diffusion MRI acquisition to clinical practice and research. *Sci. Rep.* **13**, 6010 (2023).
16. Tournier, J.-D., Calamante, F. & Connelly, A. Robust determination of the fibre orientation distribution in diffusion MRI: non-negativity constrained super-resolved spherical deconvolution. *NeuroImage* **35**, 1459–1472 (2007).
17. Takemura, H., Caiafa, C. F., Wandell, B. A. & Pestilli, F. Ensemble Tractography. *PLoS Comput. Biol.* **12**, e1004692 (2016).

18. Tournier, J. D., Calamante, F. & Connelly, A. MRtrix: Diffusion tractography in crossing fiber regions. *Int. J. Imaging Syst. Technol.* (2012) doi:10.1002/ima.22005.
